# The Mechanism of Aureusidin in Suppressing Inflammatory Response in Acute Liver Injury by Regulating MD2

**DOI:** 10.3389/fphar.2020.570776

**Published:** 2020-10-28

**Authors:** Yi Yang, Chenyang Han, Yongjia Sheng, Jin Wang, Xiaohong Zhou, Wenyan Li, Li Guo, Shuiliang Ruan

**Affiliations:** ^1^Department of Pharmacy, The Second Affiliated Hospital of Jiaxing University, Jiaxing, China; ^2^Department of Center Laboratory, The Second Affiliated Hospital of Jiaxing University, Jiaxing, China

**Keywords:** aureusidinmyeloid, differentiation protein 2, acute liver injury, inflammatory response, lipopolysaccharide, toll likes receptor

## Abstract

**Objective:**

In this study, we mainly explored the mechanism and target of the anti-inflammatory effects of Aureusidin (Aur) in acute liver injury.

**Methods:**

Lipopolysaccharide (LPS) was used to induce inflammatory injury in Kupffer cells (KCs) *in vitro*. After Aur treatment with gradient concentration, flow cytometry, propidium iodide (PI) staining, and Hoechst 33342 staining were used to detect the apoptotic level of KCs, and an enzyme-linked immunosorbent assay (ELISA) was used to detect the expression levels of inflammatory factors, including Interleukin-1β (IL-1β), Interleukin-18 (IL-18), and tumor necrosis factor alpha (TNF-α). Western blot was used to detect the expression of toll-like receptor 4 (TLR4), myeloid differentiation protein-2 (MD2), MyD88, and p-P65. Aur was labeled with biotin, followed by a pull-down assay to detect its binding with MD2. Moreover, D-GalN/LPS was used to induce acute liver injury in mice *in vitro*, followed by Aur treatment by gavage. H&E staining was used to detect the pathological changes of liver tissue, an IF assay was used to detect the expression of MD2, Western blot was used to detect the expression of relevant proteins.

**Results:**

Aur pretreatment could significantly inhibit LPS-induced KC injury, downregulate the apoptotic level, inhibit the expression of inflammatory factors, decrease the level of MDA, and downregulate the expression of MD2 in cells. Aur could inhibit the activation level of TLR4/MD2-NF-κB in a dose-dependent pattern, a high dose of Aur had a superior effect compared to low-dose Aur. In the case of MD2 deletion, the effects of Aur were suppressed. Additionally, pull-down and co-immunoprecipitation assays show that Aur can bind with the MD2 protein to inhibit the activation of TLR4/MD2-NF-κB. Results of mice experiments also showed that Aur could relieve liver injury, decrease the levels of ALT and AST, and simultaneously downregulate the levels of inflammatory factors in tissues and peripheral blood.

**Conclusion:**

We found that Aur exerted an anti-inflammatory effect by directly targeting the MD2 protein, further inhibiting the expression of TLR4/MD2-NF-κB, thereby relieving acute liver injury. Therefore, Aur might be a potential inhibitor for MD2.

## Background

The lipopolysaccharide (LPS)-mediated inflammatory response plays an important role in the pathogenesis and progression of various diseases, but without a currently effective control approach (Santos et al., 2018; Yang et al., 2018). Myeloid differentiation-protein 2 (MD2) is an important accessory protein of toll-like receptor 4 (TLR4); MD2 can assist the binding of LPS and TLR4 along with TLR4 and MyD88, thereby activating inflammatory signaling pathways (Fitzgerald et al., 2004; Schnabl et al., 2008). First, under the assistance of CD14, TLR4 and LPS form a complex with MD2. The dimerization of the complex on the cell membrane and conformational changes of the intracellular segment can cause intracellular signals, eventually leading to the conversion transcription factors, including NF-κB and AP-1 (Kurt-Jones et al., 2000), thereby inducing the expression of downstream inflammatory factors, chemokines, and adhesion factors. Macrophages are important effector cells for the TLR4 signaling-mediated inflammatory response (Oak et al., 2006; Zhang et al., 2008). Therefore, how to inhibit the inflammatory response of macrophages is the focus of research.

The liver is capable of clearing intestinal-derived bacteria and endotoxin LPS. Kupffer cells (KCs) are the main macrophages in the liver. After activation by LPS, KCs can induce local or systemic inflammation. Multiple studies have demonstrated that KCs account for over 15% of hepatic cells, playing an important role in liver immunity (Frink et al., 2007; Gong et al., 2019). The current study has also validated the role of TLR4 signaling in acute liver injury (ALI). Aureusidin (Aur) is an aurone compound extracted from the antirrhinum majus. Aur has detoxification, blood circulation, antibacterial, and anti-inflammatory effects (Nakayama, 2000), however, without clear pharmacodynamic basis and mechanism. In this study, we aimed to reveal the anti-inflammatory mechanism and pharmacodynamic targets of Aur by studying TLR4/MD2-NF-κB signaling.

## Material and Methods

### Assays on the Resistant Effects of Aur on LPS-Induced Inflammation in KCs

KC cells (Procell Life Science & Technology Co. Ltd, Wuhan China) were cultured in DMEM medium at 37 °C and 5% CO_2._ KCs in logarithmic phase were divided into a DMSO group, LPS group, and Aur group. KCs in the DMSO group were added with DMSO as control, without treatment of LPS and Aur. KCs in the LPS group were treated with 0.1 mg/L LPS to induce inflammation. KCs in the Aur group were pretreated with Aur at final concentrations of 5, 10, and 20 μM for 6 h, followed by addition of 0.1 mg/L LPS to induce inflammation. The specific detection assays were as follows:

1. Cell viability by CCK-8 assay: Cells were inoculated in 96-well plates. Three replicates were set in each group, and a blank medium was set as the control. Cell viability was measured at 0, 3, 6, and 12 h after LPS intervention. In brief, cells were added with 100μl of fresh medium in each well, incubated with 10μl of CCK-8 reagent (Beyotime Biotechnology Co., Ltd., Shanghai, China) for 2h, followed by measurement of absorbance at a wavelength of 450nm.
2. Apoptosis by flow cytometry: KCs were inoculated into 6-well plates, treated with LPS for 12 h. Afterwards, suspended cells were collected, and adherent cells were digested with 0.25% trypsin, followed by washing with PBS and centrifugation at 2000rpm for 10 min. The collected cells were resuspended with cold PBS, centrifuged again, suspended with binding buffer, and subjected to apoptotic detection by an Apoptosis Detection Kit (BD, Massachusetts, USA). In brief, cell suspension was added with 5μl of Annexin V-FITC in the dark for 5 min, incubated with 5μl of PI for 5 min, followed by flow cytometry after volume adjustment of 500μl.
3. Apoptosis detection in KCs by PI staining and Hoechst 33342 staining: KCs were stained after LPS treatment for 12 h. After discarding the medium, cells were washed with PBS twice. The Hoechst 33258 staining solution (Beyotime Biotechnology Co., Ltd.) was diluted at 1: 100 and added to the KCs. After incubation for 15 min, cells were washed with PBS twice and observed under a microscope (positive cells showed blue fluorescence). In terms of PI staining, cells were incubated with a PI staining reagent (Beyotime Biotechnology Co., Ltd.) at 1 μg/ml for 30 min, washed with PBS twice, and observed under a microscope (positive cells showed red fluorescence). The above two staining approaches were used to detect the number of apoptotic cells.
4. The expression level of inflammatory factors IL-1β, IL-18, and TNF-α by ELISA: After LPS treatment for 12 h, the culture medium was collected, centrifuged at 3000r/min, and subjected to ELISA detection using the ELISA kit instructions (shown as pg/ml).
5. SOD level by a WST assay and MDA level by a TAB assay: KCs were seeded into 6-well plates. When cells grew into the logarithmic phase, cells were treated with LPS for 12 h. Afterwards, cells were washed with cold PBS, added with lysis buffer for 30min, and centrifuged at 4 °C to collect the supernatant. The supernatant was divided into two parts. The expression of SOD was assessed by a WST kit (Nanjing Jiancheng Bioengineering Institute, Nanjing, China) (shown as U/mg), and the expression of MDA was determined by a TAB kit (Nanjing Jiancheng Bioengineering Institute) (shown as μmol/mg).
6. Detection of MD2 expression by an immunofluorescence (IF) assay: A cell slide was used for IF staining. In brief, coverslips were placed in a 6-well plate. After cell adherence, cells were treated with LPS for 12 h, followed by fixation using freshly prepared 4% paraformaldehyde (PFA) for 10 min. Afterwards, cells were washed with PBS three times, permeabilized with 0.2% Triton X-100 for 10 min, blocked with 2% BSA for 30 min, incubated with MD2 monoclonal antibody (dilution 1:300) (Abcam, Massachusetts, the USA) at room temperature for 1 h, washed with PBS three times, subsequently reacted with the IgG antibody (Abcam, Massachusetts, the USA), and was stained with 0.5μg/ml of a DAPI staining reagent (Solarbio, Beijing, China). After washing with PBS twice, slices were mounted and observed under fluorescent microscope.
7. Detection of the protein expression level of TLR4, MD2, MyD88, and p-P65 by Western blot: KC cells were seeded in 6-well plates. After cell adherence, cells were treated with LPS for 12 h, washed with PBS twice, and centrifuged. The collected tissues and cells were ground with liquid nitrogen, lysed in RIPA lysate (Beyotime Biotechnology Co., Ltd.) (1.0ml) on ice for 30 min, and centrifuged at 10,000g for 15 min. The protein supernatant was collected and quantified. The protein sample was transferred to the PVDF membrane using 300mA constant current for 0.5-2h, blocked with 5% skimmed milk powder for 2 h, and incubated with primary monoclonal antibodies against TLR4, MD2, MyD88, and p-P65 (dilution 1:500 in TBST). After washing with TBST twice, the membranes were incubated with a horseradish peroxidase (HRP)-conjugated goat anti-rabbit secondary antibody (dilution 1:20000, Abcam, USA). Afterwards, the chemiluminescence method was used for visualization, and the Image Pro-Plus 6.0 software was used to analyze the optical density. GAPDH was used as the internal control. The results were shown as the comparison of the optical density values of the target protein with the internal control.

### Assays on the Anti-Inflammatory Effect of Aur in KCs With MD2 Knockout

CRISPR-Cas9 was used to knock out MD2 in KC (monoclonal, KC-MD2^-/-^), followed by validation by quantitative PCR (qPCR) and Western blot. The primer sequences of MD2 were as follows: Forward: 5’-ACCTCGAGGGAAGAGTCTGATGATCAGTTACTG-3 ‘; Reverse: 5’-GCGGTACCCTAATTTGAATTAGGTTGGTGTAGG-3’ (564bp). The MD2-sgRNA sequence for CRISPR-Cas9 was as follows: TTCCGGCGCGCCGAGTCCTTAGG. The protein and mRNA expression of MD2 in KC-MD2^-/-^ were verified by Western blot and qPCR.

KC-MD2^-/-^ cells were divided into DMSO, LPS, and Aur groups. The DMSO group was the control group, cells in the LPS group were treated with 0.1 mg/L LPS to induce inflammation, while cells in the Aur group were pretreated with Aur at a final concentration of 20 μM for 6 h, followed by treatment with 0.1 mg/L LPS for 12 h to induce inflammation. Afterwards, the following assays were performed accordingly.

### Validation of Aur Targeting MD2

Virtual docking was adopted to investigate the binding between Aur and MD2. The 3D structure of Aur was obtained from the pubchem website (https://pubchem.ncbi.nlm.nih.gov/). The receptor protein MD2 (PDB ID: 2E59) was obtained from the Protein Data Bank (http://www.rcsb.org/pdb) database, followed by dehydration and de-ligand of the receptor protein using the PYMOL 2.3.4 software. The MGLTools software was used to hydrogenate and calculate the charge of the two receptor proteins. The appropriate box center (center_x = -0.622, center_y = 21.731, center_z = 13.206) and box grid (size_x = 40, size_y = 40, size_z = 40) parameters of the MD2 receptor protein were set, including the possible binding active pocket sites of small molecule ligands. Afterwards, the MD2 receptor protein and Aur were subjected to molecular docking (AutoDock Vina 1.1.2) to obtain 20 conformations. PyMOL was further used to visualize the hydrogen bonding between the receptor protein and the ligand small molecule of the optimal conformation (-9.4kcal/mol). We found that Aur could target MD2, which was further validated.

#### Validation of the Binding of Aur With MD2 Protein Using bis-ANS Fluorescence Spectroscopy

First, bis-ANS fluorescence detection was performed. In brief, 95 μl of PBSr and 5 μl of bis-ANS solution were added to the cuvette, followed by fluorescence detection using a microplate reader (excitation wavelength: 385nm, emission wavelength: 430-550nm). Afterwards, 90 μl of PBS, 5 μl of 0.1 mM bis-ANS solution, and 5 μl of 0.1 μM rhMD2 protein (Abcam Corporation, Massachusetts, the USA) were further added to the cuvette and mixed, followed by fluorescence detection (excitation wavelength: 385 nm, emission wavelength: 430-550nm). This approach was used to assess the fluorescence absorption characteristics of MD2. Finally, we validated the effect of Aur on MD2. Briefly, 87.5 μl of PBS, 5 μl of 0.1 mM bis-ANS solution, 5 μl of 0.1 μM rhMD2 protein, and Aur (concentration: 2.5-5-10-20μM) were added to the cuvette, and incubated at room temperature for 5 min, followed by fluorescence detection. The Graphpad Prism 8.0 software was used to plot curves.

#### Detection of the Binding of Aur With MD2 by Co-Immunoprecipitation (Co-IP)

KCs were seeded into 6-well plates, treated with DMSO, LPS, and Aur accordingly, and incubated for 4 h. After washing with PBS, cells were lysed in 120 μl of lysate on ice, collected and transferred into EP tubes, and centrifuged. The collected supernatant was transferred to a new EP tube, followed by protein quantification by a BCA assay. Afterwards, the protein sample was incubated with an anti-TLR4 monoclonal antibody (2 μL antibody/200 μg protein sample) at 4 °C overnight, followed by incubation with protein G agarose at 4 °C on a shaker for 4 h. The supernatant was collected and washed with cold PBS four times, followed by Western blot to detect the expression of TLR4 and MD2.

#### Biotin-Aur-MD2 Pull-Down Assay

The SUMO-labeled MD2 protein was expressed in E. coli overnight. Afterwards, the supernatant was diluted at 1:50, induced with β-D-thiogalactopyranoside, followed by detection of OD600 at 0.7-0.8. Cells were suspended in Tris-HCl and NaCl, collected, sonicated, centrifuged, and purified with a Ni column. On the column, the His-SUMO-MD2 protein was washed twice with 500 mM imidazole, the His-SUMO tag was cleaved with SUMO protease (ULP-1), followed by purification with the Ni column. The purified protein was validated by SDS-PAGE and Coomassie blue staining. The 15μg of recombinant protein was combined with biotin-labeled Aur (Biotin-Aur) for pull-down with streptavidin beads (Sigma Massachusetts, USA). Beads were blocked in 5% BSA in Tris buffer, while the protein was precleared with beads that were subsequently discarded. After incubation with 50 μM Biotin-Aur, beads were washed three times with Tris buffer, followed by boiling with the buffer and β-mercaptoethanol. For MD2 pull-down, recombinant G protein magnetic beads were incubated with the MD2 antibody (Abcam, Massachusetts, USA). After washing with Tris buffer, Western blot was used to detect the expression of MD2, followed by a HRP-conjugated anti-biotin antibody to detect biotin (CST, Boston, USA).

#### Interventional Effect of Aur on ALI in Mice

Animal experiments were reviewed and approved by the ethics committee of Jiaxing second hospital, the number: 2019-AE-13122. Clean-grade C57BL/6 mice were randomly divided into the Con group, D-GalN/LPS group, and Aur group. Before constructing the ALI mouse model, mice in the Aur group were administered with Aur 20mg/kg by gavage daily for seven consecutive days, while mice in the Con group and D-GalN/LPS group were administered with the equal volume of normal saline by gavage. Twenty-four hours after the last administration, mice in the D-GalN/LPS group and the Aur group were intraperitoneally injected with D-GalN (Sigma, Massachusetts, USA) (1000 mg/kg) and LPS (Sigma, Massachusetts, USA) (10 μg/kg) for the construction of the ALI model.

##### The Pathological Changes of the Liver Tissues of Mice Using H & E Staining

After injection of LPS/D-GalN for 72 h, mice were suffocated with carbon dioxide. Afterwards, the liver tissues were resected, embedded into paraffin, and serially cut into 4μm sections. The slices were dewaxed by xylene, dehydrated with 100%, 95%, and 80% gradient-concentration ethanol, rinsed under tap water for 2 min, stained with hematoxylin for 3 min, rinsed under tap water for 2 min, treated with 1% hydrochloric acid alcohol for 2 s, rinsed under tap water for 2 min, reacted with 1% ammonia water for 2 0s, stained with 0.5% eosin alcohol for 10 s, dehydrated with gradient-dehydration alcohol, made transparent by xylene, and sealed by neutral gum. Finally, the pathological changes of the liver tissues were observed under a light microscope.

##### Detection of the Expression of ALT, AST, and Inflammatory Factors Using Relevant Kits

The detection of ALT and AST was performed by UV colorimetry (Nanjing Jiancheng Bioengineering Institute, Nanjing, China). The peripheral blood of mice was centrifuged according to the manufacturer’s instructions. The detection of inflammatory factors, including IL-1β, IL-18, and TNF-α in peripheral blood and liver tissue was performed by an ELISA kit (Nanjing Jiancheng Bioengineering Institute, Nanjing, China). Peripheral blood was centrifuged to collect serum, and liver tissue was cut using sterile surgical scissors and ground with liquid nitrogen, followed by lysis in 1.0ml of RIPA lysate on ice for 30 min. After centrifuging at 10,000g for 15 min, the supernatant was quantified according to the manufacturer’s instructions. The expression of AST and ALT was shown as U/L, and the expression of inflammatory factors, including IL-1β, TNF-α, and IL-18 was shown as pg/ml.

##### Detection of MD2 Expression in Mouse Liver by Immunohistochemistry (IHC) Staining

The paraffin-embedded liver tissue sections were serially cut into 4μm-thick slices, baked at 60 °C for 2 h, dewaxed in xylene three times (5 min each), immersed in absolute ethanol for 5 min, immersed in 95% ethanol twice (2 min each), immersed in 85% ethanol for 2 min, rinsed under tap water for 5 min, and rinsed under distilled water for 3 min. The slices were subsequently placed in 0.01mol/L citrate buffer (PH = 6.0) and heated at 98 °C for 20 min in a microwave oven for antigen retrieval, cooled at room temperature for 30 min, and rinsed under distilled water. Afterwards, the slices were incubated with 3% hydrogen peroxide at room temperature for 10 min to eliminate endogenous peroxidase and blocked with 2% bovine serum albumin (BSA) at 37 °C for 30 min to prevent non-specific binding. After discarding BSA, the slices were incubated with a primary antibody against MD2 (dilution ratio 1: 300, Abcam, USA) at 37 °C for 2 h. After washing with TBS three times (5 min each), the slices were reacted with the corresponding secondary antibody at 37 °C for 15 min, incubated and peroxidase-labeled with streptomycin (Abcam, USA) for 15 min, and rinsed with PBS three times (5 min each). Each slice was added with a freshly prepared DAB solution for visualization (DAKO, Denmark), and was observed under microscope for reaction termination. The slices were rinsed thoroughly under tap water, counterstained with hematoxylin, and mounted. In terms of the negative control, the primary antibody was replaced by TBS. All slices were photographed under the Olympus-BX51 upright microscope with the Olympus-DP72 image acquisition system and CRi Nauance multi-spectral imaging system (Cambridge Research & Instrumentation, USA).

##### Detection of the Expression of TLR4, MD2, MyD88, and p-P65 by Western Blot

100 mg of liver tissue was cut with sterile surgical scissors, ground with liquid nitrogen, lysed in 1.0 ml of RIPA lysate (Beyotime Biotechnology Co., Ltd.), on ice for 30 min, centrifuged at 10,000 g for 15 min, followed by protein quantification of the supernatant. Finally, Western blot was performed to detect the protein expression accordingly.

### Statistical analysis

Data are expressed as mean ± standard deviation $\left(\bar{x}\pm s\right)$ . Differences among groups were tested by one-way ANOVA. Comparisons between two groups were performed by an unpaired Student’s t-test. A value of P <0.05 was considered statistically different.

## Results

### Aur Could Inhibit the LPS-Induced Inflammatory Response and Injury in KCs

**Figure 1:**
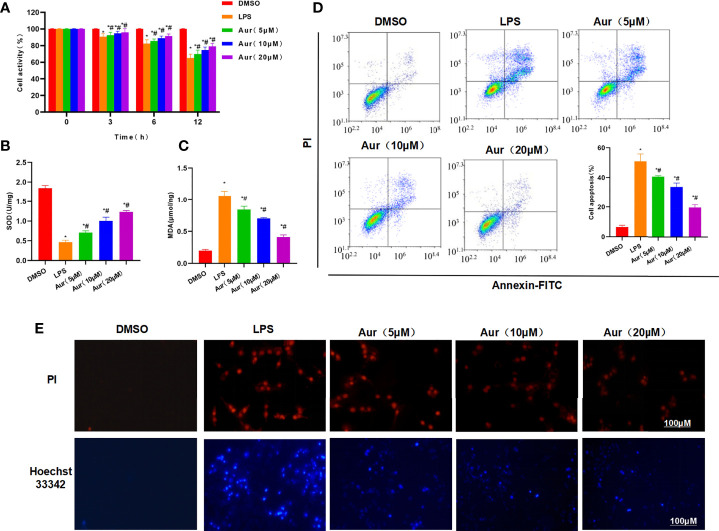
Effect of Aur on LPS-induced KC injury. **(A)**: Effect of Aur on cell viability (n = 3): The cell viability in the LPS group was significantly more decreased than the DMSO group; while Aur treatment could suppress the decrease in viability, which was significantly different from the LPS group. In addition, the pairwise comparison was also statistically significant. Comparison with DMSO, ^*^P <0.05; comparison with LPS group, ^#^P <0.05. **(B, C)**: Effect of Aur on SOD and MDA (n = 3): The SOD level was downregulated and the MDA level was upregulated in the LPS group, which were significantly different from the DMSO group. Aur treatment could decrease the MDA level and increase the SOD level in a dose-dependent pattern. Comparison with DMSO, ^*^P <0.05; comparison with LPS group, ^#^P <0.05. **(D)**: Apoptosis assay (n = 3): The apoptotic level was low in the DMSO group, LPS could significantly increase the apoptotic level, and Aur could downregulate the apoptotic level. Comparison with DMSO, ^*^P <0.05; comparison with LPS group, ^#^P <0.05. **(E)**: PI staining and Hoechst 33342 staining (n = 3): Positive cells were barely detectable in the DMSO group, which were significantly upregulated in the LPS group. While the number of positive cells was downregulated in the Aur group, indicating the suppressed apoptosis.

**Figure 2:**
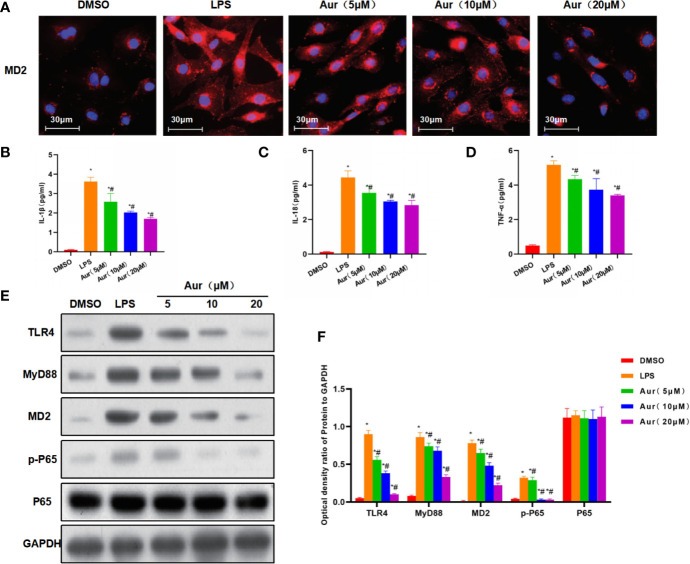
The mechanism of the inhibitory roles of Aur on inhibiting KC injury. **(A)**: IF staining of MD2 protein (n = 3): The expression level of MD2 was relatively low in the DMSO group, which was significantly increased in the LPS group, with higher increased fluorescence intensity than the DMSO group. While Aur treatment could inhibit the expression of the MD2 protein, the fluorescence intensity was downregulated, which were significantly different from LPS treatment. **(B–D)**: Detection of inflammatory factors (n = 3): The levels of inflammatory factors, including IL-18, IL-1β, and TNF-α were relatively low in the DMSO group, which were significantly upregulated by LPS treatment; while Aur treatment could suppress the levels of inflammatory factors. Comparison with DMSO, ^*^P <0.05; comparison with LPS group, ^#^P <0.05. **(E, F)**: The expression of key proteins of the TLR4/MD2-NF-κB signal (n = 3): The expression levels of TLR4, MyD88, MD2, and p-P65 were significantly more upregulated after LPS induction than DMSO treatment, while the expression of P65 was not significantly changed. Aur treatment could downregulate the expression of TLR4, MyD88, MD2, and p-P65 in a dose-dependent manner, while the expression of P65 was not significantly changed. Comparison with DMSO, ^*^P <0.05; comparison with LPS group, ^#^P <0.05.

LPS can induce injury and the release of inflammatory factors in KCs. LPS induction could significantly decrease the viability of KCs, which could be significantly suppressed by Aur in a dose-dependent pattern compared with the LPS group (P <0.05) (**Figure 1A**). An apoptosis assay also showed that Aur can inhibit the apoptosis of KCs (**Figure 1D**). The detection of SOD and MDA revealed that LPS can decrease the level of SOD and increase the level of MDA; while Aur can increase the SOD level and decrease the MDA level, which were significantly different compared with the LPS group, with significantly superior effects in the high-dose group than the low-dose group (P <0.05) (**Figures 1B, C**). PI staining and Hoechst 33342 staining also showed that LPS treatment could significantly induce the apoptosis of KCs, with an upregulated level of positive cells; the number of positive cells was significantly downregulated in the Aur group than the LPS group (P <0.05) (**Figure 1E**). IF staining of MD2 showed that the expression level of MD2 was low in the DMSO group. LPS treatment could significantly upregulate the expression of MD2, and Aur treatment could downregulate the level of MD2, with decreased fluorescence intensity (**Figure 2A**). In terms of the expression of inflammatory factors, Aur treatment could significantly inhibit the release of LPS-induced inflammatory factors. The levels of IL-18, IL-1β, and TNF-α in the Aur group were significantly lower than those in the LPS group in a dose-dependent pattern (**Figures 2B–D**). The detection of key proteins in the TLR4 signal also showed that LPS could activate the TLR4 signal and upregulate the expression level of TLR4, MyD88, MD2, and p-P65, but not P65; while Aur treatment could downregulate the protein level (**Figures 2E, F**).

### The Effect of Aur on Inflammatory Response and Injury in KCs After MD2 Knockout

**Figure 3:**
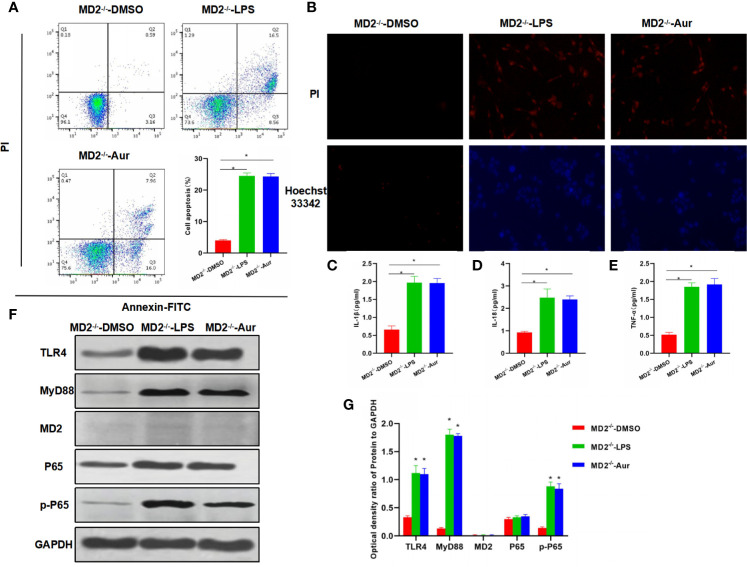
Effects of Aur on KC injury after MD2 knockout. **(A)**: Detection of apoptotic level (n = 3): There was no significant apoptosis in the MD2^-/–^DMSO group, while the apoptotic level was significantly more increased in the MD2^-/–^LPS and MD2^-/–^Aur groups than the MD2^-/–^DMSO group, however, without significant difference between groups. Comparison between groups, ^*^P <0.05. **(B)**: PI staining and Hoechst 33342 staining (n = 3): There were no positive cells in the MD2^-/–^DMSO group, without significant apoptosis. The number of positive cells was not significantly different between the MD2^-/–^LPS and MD2^-/–^Aur groups. **(C–E)**: Expression of inflammatory factors (n = 3): The expression level of inflammatory factors was not significantly different between the MD2^-/–^LPS and MD2^-/–^Aur groups, which was significantly more increased than the MD2^-/–^DMSO group. Comparison between groups, *P <0.05. **(F, G)**: Detection of the protein expression level (n = 3): The expression level of TLR4, MyD88, MD2, and p-P65 was relatively low in the MD2^-/–^DMSO group, which was significantly upregulated in the MD2^-/–^LPS and MD2^-/–^Aur groups, however, without statistical significance between groups. Comparison between groups, *P <0.05.

Next, we aimed to investigate whether Aur affected the functions of MD2 after MD2 knockout (KC-MD2^-/-^ cells). As a result, an apoptosis assay showed that there was no significant apoptosis in the MD2^-/–^DMSO group, while the apoptotic level between MD2^-/–^LPS and MD2^-/–^Aur was not significantly different, which was significantly higher than the MD2^-/–^DMSO group (**Figure 3A**). PI staining and Hoechst 33342 staining also showed no significant change of the number of positive cells between the MD2^-/–^LPS and MD2^-/–^Aur groups (**Figure 3B**). The detection of the expression of inflammatory factors revealed that the expression level of inflammatory factors was relatively low in the MD2^-/–^DMSO group, while the expression of inflammatory factors was upregulated in the MD2^-/–^LPS and MD2^-/–^Aur groups, however, without significant differences between the groups (**Figures 3C–E**). A Western blot assay also revealed that the protein expression of Aur was not significantly changed after MD2 knockout, indicating that Aur might change the level of MD2, but not other proteins, such as TLR4 (**Figures 3F–G**).

### The Inflammatory Mechanism of Aur Targeting MD2

**Figure 4:**
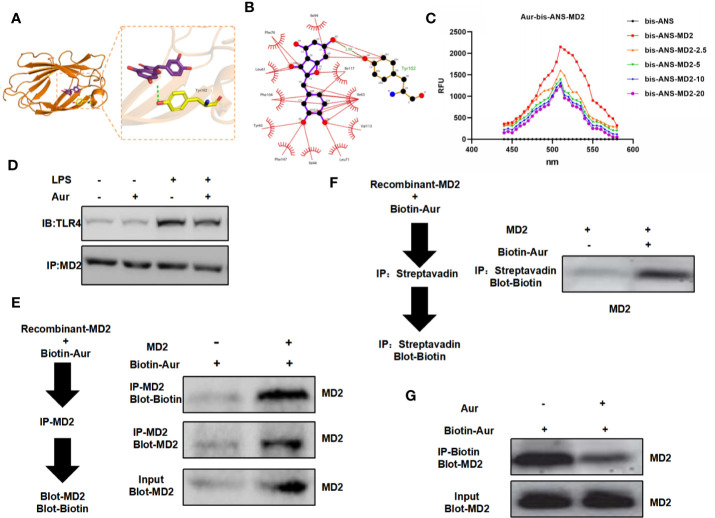
Validation of the targeted binding correlation between Aur and MD2 protein. **(A, B)**: The virtual docking of the binding between Aur and MD2. **(C)**: A bis-ANS assay showed that Aur could decrease the fluorescence intensity in a dose-dependent manner. **(D)**: A co-IP assay showed no significant binding between Aur and TLR4, but that Aur could bind with MD2. **(E, F)**: A pull-down assay revealed that Aur could bind with MD2. **(G)**: A competitive binding assay demonstrated that large doses of Aur could compete with Biotin-Aur and decrease the binding level of MD2.

The binding mode between the Aur ligand small molecule and the receptor protein MD2 was as follows: the amino acid residue Tyr102 formed a hydrogen bond with the Aur ligand small molecule, the amino acid residues Phe76, Leu61, Phe104, Tyr65, Phe147, Ile44, Leu71, Val113, Ile63, Ile117, and Ile94 showed a hydrophobic interaction with the Aur ligand small molecule, and Aur could bind to the MD2 protein (**Figures 4A, B**). A bis-ANS assay showed that Aur could bind to the MD2 protein in a dose-dependent manner (**Figure 4C**). A co-IP assay revealed that Aur could bind to the MD2 protein, which was not associated with the TLR4 protein (**Figure 4D**). After Biotin-Aur labeling, the pull-down assay showed that MD2 could directly bind to Aur, and Aur could compete with Biotin-Aur, also indicating that MD2 was the target protein of Aur (**Figures 4E–G**).

### The Interventional Effect and Mechanism of Aur on Mice With Liver Injury

**Figure 5:**
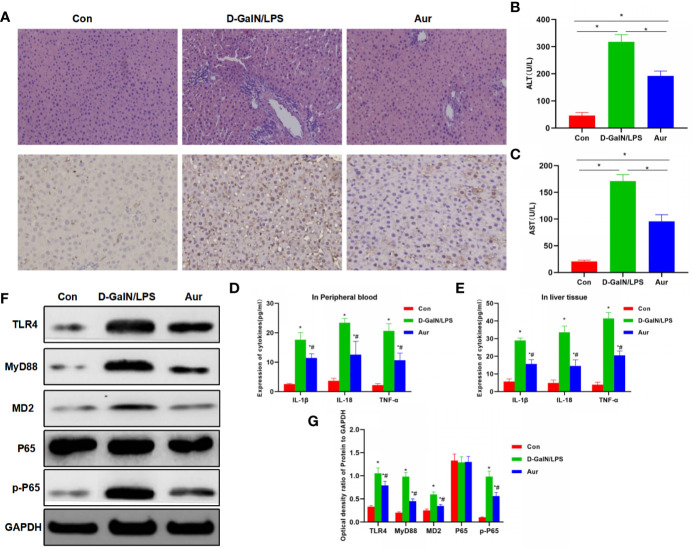
Effect of Aur on ALI in mice. **(A)**: Effects of Aur on tissue pathology and MD2 expression of liver in mice (n = 5): There was no significant liver injury in mice from the Con group, with relatively low expression of MD. In the LPS/D-GalN group, there was an inflammatory response, tissue edema, and significantly increased expression of MD2 in the liver tissue, while the tissue inflammatory response was relieved and MD2 expression was downregulated in the Aur group. **(B, C)**: Alteration of ALT and AST expression in mice (n = 10): The expression of ALT and AST was relatively low in the Con group, which was upregulated in the LPS/D-GalN group, but was downregulated in the Aur group. Comparison between groups, ^*^P <0.05. **(D, E)**: Changes in the expression of inflammatory factors in the peripheral blood and liver tissues of mice (n = 10): The expression of inflammatory factors, including IL-18, IL-1β, and TNF-α was relatively low in the Con group, which was upregulated in the LPS/D-GalN group, but was downregulated in the Aur group. Comparison with Con, ^*^P <0.05; comparison with LPS/D-GalN group, ^#^P <0.05. **(F, G)**: Detection of protein expression in liver tissue from mice (n = 5): The expression of TLR4, MyD88, MD2, and p-P65 was relatively low in the Con group, which was significantly upregulated in the LPS/D-GalN group, and was inhibited in the Aur group. Comparison with Con, ^*^P <0.05; comparison with LPS/D-GalN group, ^#^P <0.05.

The liver tissue of mice from the LPS/D-GalN group showed obvious pathological changes, obvious inflammatory response in liver tissue, apoptosis, and tissue edema, which was significantly different from the Con group. However, after Aur pretreatment in the Aur group, tissue lesions were significantly attenuated, and inflammatory response and tissue edema were significantly relieved. IHC staining showed that the expression of MD2 was relatively low in the Con group, along with the suppressed expression of MD2 in the Aur group (**Figure 5A**). The detection of AST and ALT showed that the levels of ALT and AST were relatively low in the Con group, which was significantly upregulated in the LPS/D-GalN group, indicating the significant liver injury in mice. The expression of AST and ALT was significantly downregulated in the Aur group, which was also significantly different from the LPS/D-GalN and Con groups (P <0.05) (**Figures 5B, C**). The detection of inflammatory factors also showed that the expression of inflammatory factors, including IL-18, IL-1β, and TNF-α in peripheral blood and liver tissues was relatively lower in the Con group, which was significantly upregulated in the LPS/D-GalN group, while Aur treatment could suppress the expression of inflammatory factors in the peripheral blood and tissues (**Figures 5D, E**). In terms of the expression of key proteins in liver tissue, the expression levels of TLR4, MyD88, MD2, and p-P65 was relatively low in the Con group, which was significantly upregulated in the LPS/D-GalN group than the Con group. However, the expression was barely detectable in the Aur group, which was lower than the LPS/D-GalN group (**Figures 5F, G**).

## Discussion

ALI is a common liver disease; and the pathogenesis of endotoxin-induced liver injury is complex, where KCs play an important role in the pathogenesis and development of liver injury (Xu et al., 2008). The function of KCs is associated with the activation state. KCs are a typical type of mononuclear-phagocytic cells, which can mediate inflammatory reactions. Under special liver microenvironments, KCs can receive LPS-derived signals and interact with adjacent cells (Gong et al., 2018). LPS and D-GalN are the main chemicals that induce liver injury. LPS signal recognition and free LPS are transported in the form of a complex. LPS forms a complex receptor assisted by TLR4 and other accessory molecules CD14 and MD2. With the transmembrane characteristics of TLR4 (Fisher et al., 2013; Saikia et al., 2017), in addition, MyD88-dependent pathways can enrich IL-1R through the adaptor TIRAP, induce TRAF6 and activate the TAK1/TAB1/2/3 complex, induce IκB degradation, and NF-κB translocation, further promoting the transcription of inflammatory factors (Sacre et al.; Nagpal et al.). Therefore, in the canonical signal, the mediation of TLR4 is crucial. MD2 is a complex that binds to TLR4, mediates the LPS-induced inflammatory response, playing an important role in sepsis and malignant tumors. Studies have found that TLR4/MD2 receptors can cause the release of downstream inflammatory factors and initiate inflammatory response after receiving upstream signals. Meanwhile, MD2 can also activate the NF-kB signal (Han et al., 2017). In the study of MD2, MyD88 has also been revealed to assist the role of TLR4/MD2, playing an important role in signal transmission and activation. In the study of upstream regulatory signals, HMGB1 has been revealed as one of the important receptors that regulates the activation of TLR4, and LPS can activate the inflammatory response through this target (Deniz et al., 2018; Wu et al., 2018; Xu et al., 2018; Aba et al., 2020; Macías-Rodríguez et al., 2020).

Aurones are a kind of flavonoid compounds. At present, there has been no study on the pharmacological effect of Aur. In this study, LPS was used to induce inflammatory injury of KCs. KC cells play an important role in liver injury, especially in the inflammatory response. LPS treatment could induce cell injury and the expression of inflammatory factors in KCs. We aimed to intervene with Aur and found that Aur treatment could significantly attenuate the inflammatory injury of KCs. In consideration of the important role of TLR4/MD2 signaling in inflammation, we found that Aur treatment could decrease the TLR4 signal, especially in MD2 expression. Virtual docking was further adopted, revealing the perfect binding sites between MD2 and Aur. Therefore, we speculated that MD2 was the main target of Aur. To this end, MD2 was knocked out. After high-dose Aur intervention, MD2 deletion could be the effect of Aur, and Aur intervention had no significant effect on cell injury and the expression of inflammatory factors, also suggesting that MD2 may be a direct target of Aur. Co-IP and pull-down assays also showed that Aur could directly bind to MD2, fully proving that MD2 was the main target of Aur. In the study of the mouse liver injury model, Aur treatment could inhibit liver injury in mice, attenuate the pathological changes of liver tissues, downregulate the expression of inflammatory factors in peripheral blood and liver tissues, and decrease the expression of MD2 in tissues. Both *in vivo* and *in vitro* assays validated that Aur exerted a good anti-inflammatory effect on liver injury.

Collectively, Aur can target MD2 protein and inhibit the activation of the TLR4/MD2 signal, playing an important therapeutic role in ALI. As a potential small molecule, Aur is promising for applications in treating inflammatory diseases.

## Conclusion

In this study, we found that Aur exerted an anti-inflammatory effect by directly targeting the MD2 protein, further inhibiting the expression of TLR4/MD2-NF-κB, thereby relieving acute liver injury. Therefore, Aur might be a potential inhibitor for MD2.

## Data Availability Statement

The raw data supporting the conclusions of this article will be made available by the authors, without undue reservation, to any qualified researcher.

## Ethics Statement

The animal study was reviewed and approved by Jiaxing University. Written informed consent was obtained from the owners for the participation of their animals in this study.

## Author Contributions

YY, CH: Design and operation of the experiment. YS, JW: Operation of the animal experiment and data processing. XZ, WL: Collection of clinical samples and detection of inflammatory factors. LG, SR: The proposal of the subject, the design of the experimental process, and guidance for the whole process. All authors contributed to the article and approved the submitted version.

## Funding

The ZheJiang Provincial Natural Science Foundation [LYY20H280005].

## Conflict of Interest

The authors declare that the research was conducted in the absence of any commercial or financial relationships that could be construed as a potential conflict of interest.

## Abbreviations

SOD, superoxide dismutase; PI, propidium iodide; MDA, malondialdehyde; ELISA, enzyme linked immunosorbent assay; WST, water-soluble tetrazolium salt; TLR4, toll-like receptor 4; MD2, myeloid differentiation protein-2; LPS, lipopolysaccharide; ALT, alanine aminotransferase; AST, aspartate aminotransferase.
