# Corrigendum: The Mechanism of Aureusidin in Suppressing Inflammatory Response in Acute Liver Injury by Regulating MD2

**DOI:** 10.3389/fphar.2021.802304

**Published:** 2021-12-06

**Authors:** Yi Yang, Chenyang Han, Yongjia Sheng, Jin Wang, Xiaohong Zhou, Wenyan Li, Li Guo, Shuiliang Ruan

**Affiliations:** ^1^ Department of Pharmacy, The Second Affiliated Hospital of Jiaxing University, Jiaxing, China; ^2^ Department of Center Laboratory, The Second Affiliated Hospital of Jiaxing University, Jiaxing, China

**Keywords:** aureusidinmyeloid, differentiation protein 2, acute liver injury, inflammatory response, lipopolysaccharide, toll likes receptor

In the original article, there was a mistake in Figure 1E as published. We used wrong atypical images in the layout and did not mark the magnification. The corrected Figure 1 appears below.

**FIGURE 1:**
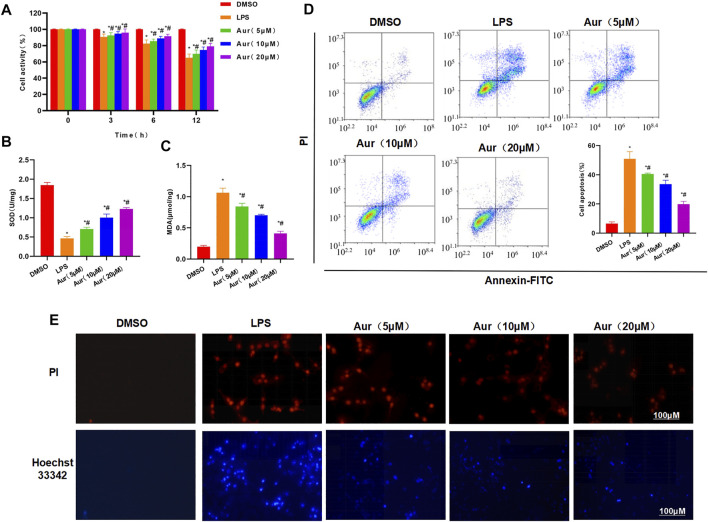
Effect of Aur on LPS-induced KC injury. **(A)** Effect of Aur on cell viability (n = 3). The cell viability in the LPS group was significantly more decreased than the DMSO group; while Aur treatment could suppress the decrease in viability, which was significantly different from the LPS group. In addition, the pairwise comparison was also statistically significant. Comparison with DMSO, *p < 0.05; comparison with LPS group, ^#^ *p* < 0.05. **(B,C)** Effect of Aur on SOD and MDA (n = 3). The SOD level was downregulated and the MDA level was upregulated in the LPS group, which were significantly different from the DMSO group. Aur treatment could decrease the MDA level and increase the SOD level in a dose-dependent pattern. Comparison with DMSO, *p < 0.05; comparison with LPS group, ^#^ *p* < 0.05. **(D)** Apoptosis assay (n = 3). The apoptotic level was low in the DMSO group, LPS could significantly increase the apoptotic level, and Aur could downregulate the apoptotic level. Comparison with DMSO, *p < 0.05; comparison with LPS group, ^#^ *p* < 0.05. **(E)** PI staining and Hoechst 33342 staining (n = 3). Positive cells were barely detectable in the DMSO group, which were significantly upregulated in the LPS group. While the number of positive cells was downregulated in the Aur group, indicating the suppressed apoptosis.

The authors apologize for this error and state that this does not change the scientific conclusions of the article in any way. The original article has been updated.

