# Supplementary figures and images for: The Mechanism of Aureusidin in Suppressing Inflammatory Response in Acute Liver Injury by Regulating MD2
Source: Front Pharmacol. 2020 Oct 28;11:570776. doi: 10.3389/fphar.2020.570776 (PMC7655772; doi:10.3389/fphar.2020.570776)

## Slide 1
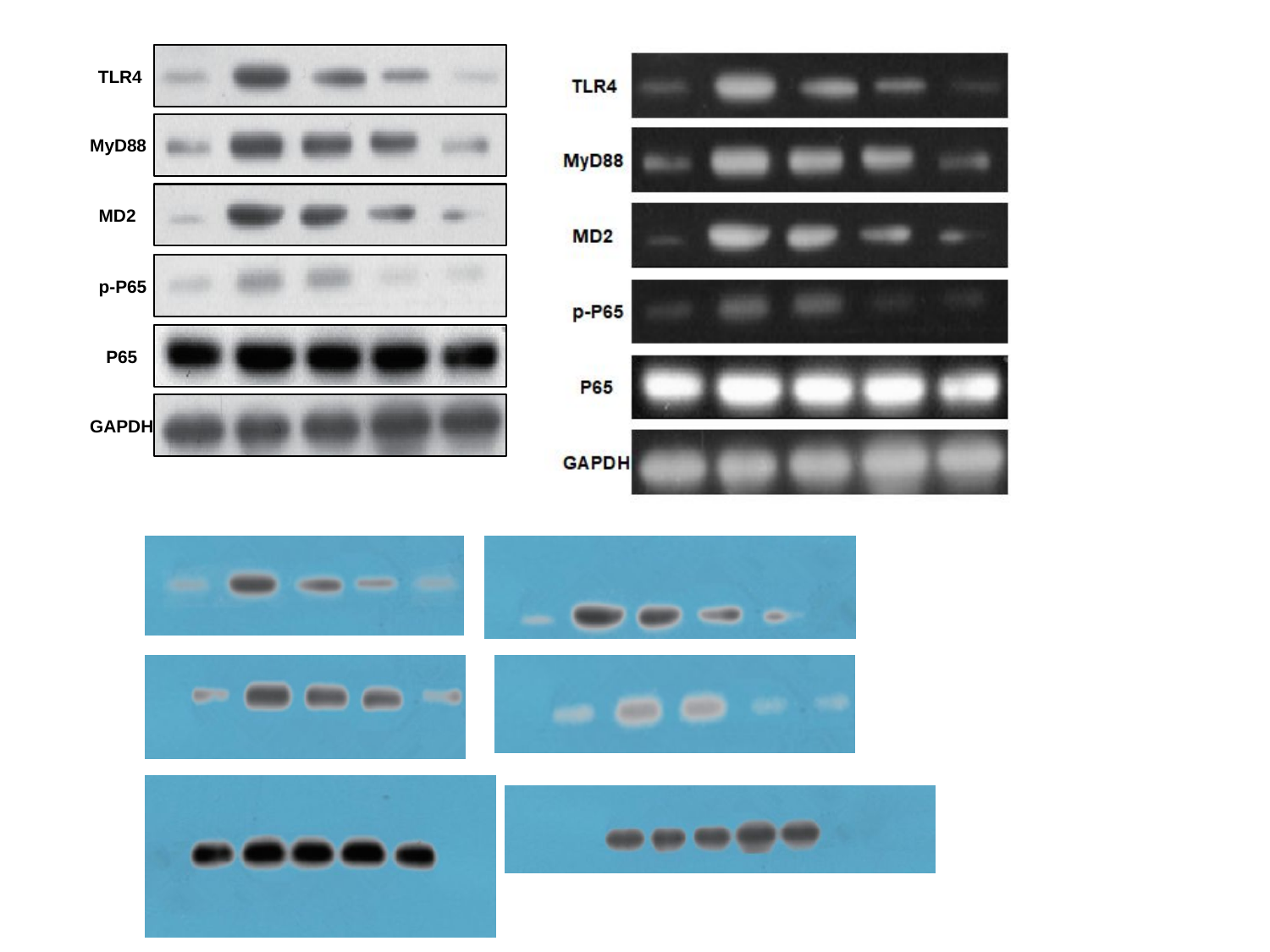

TLR4
MyD88
MD2
p-P65
P65
GAPDH

## Slide 2
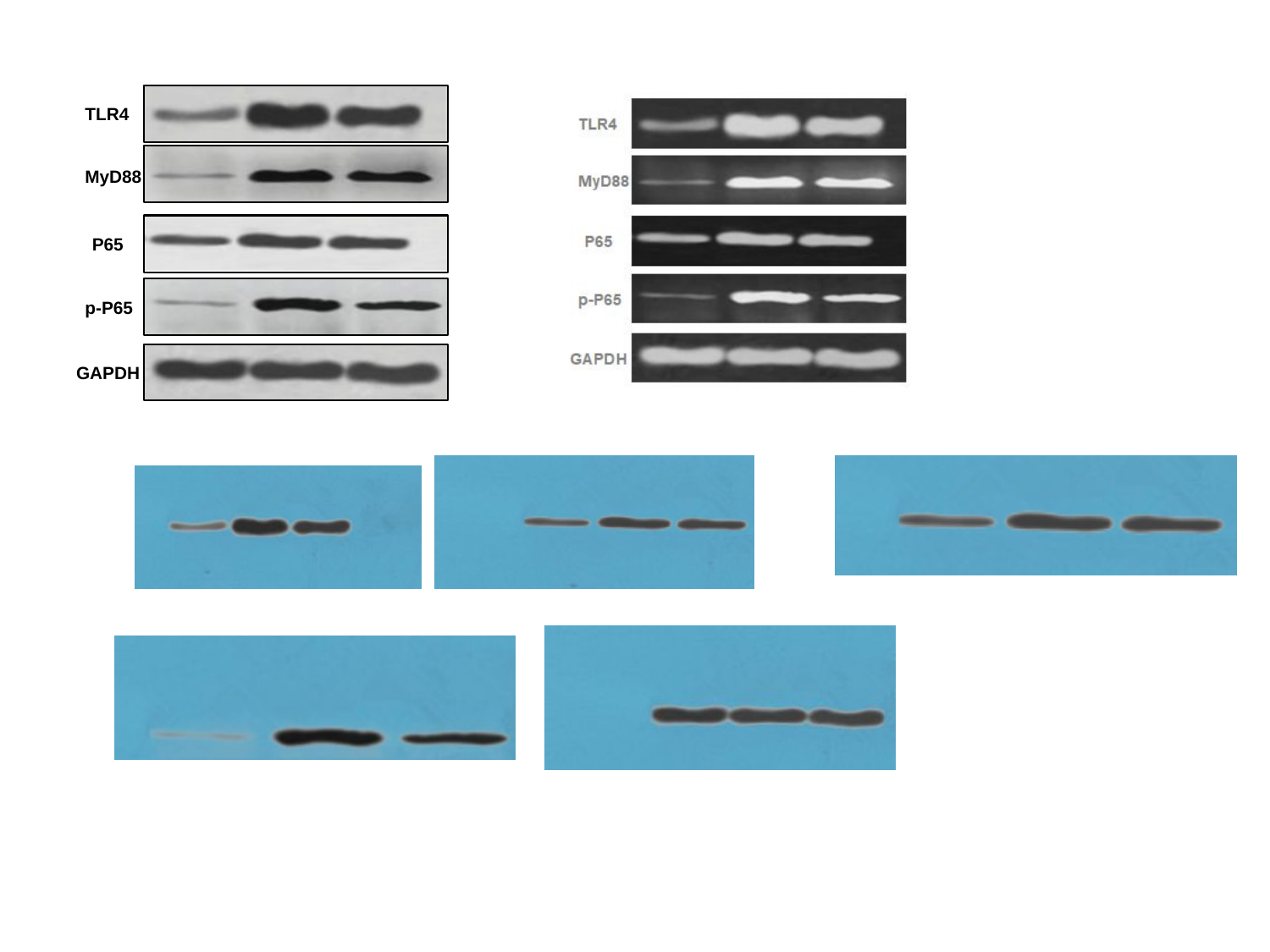

TLR4
MyD88
P65
p-P65
GAPDH

## Slide 3
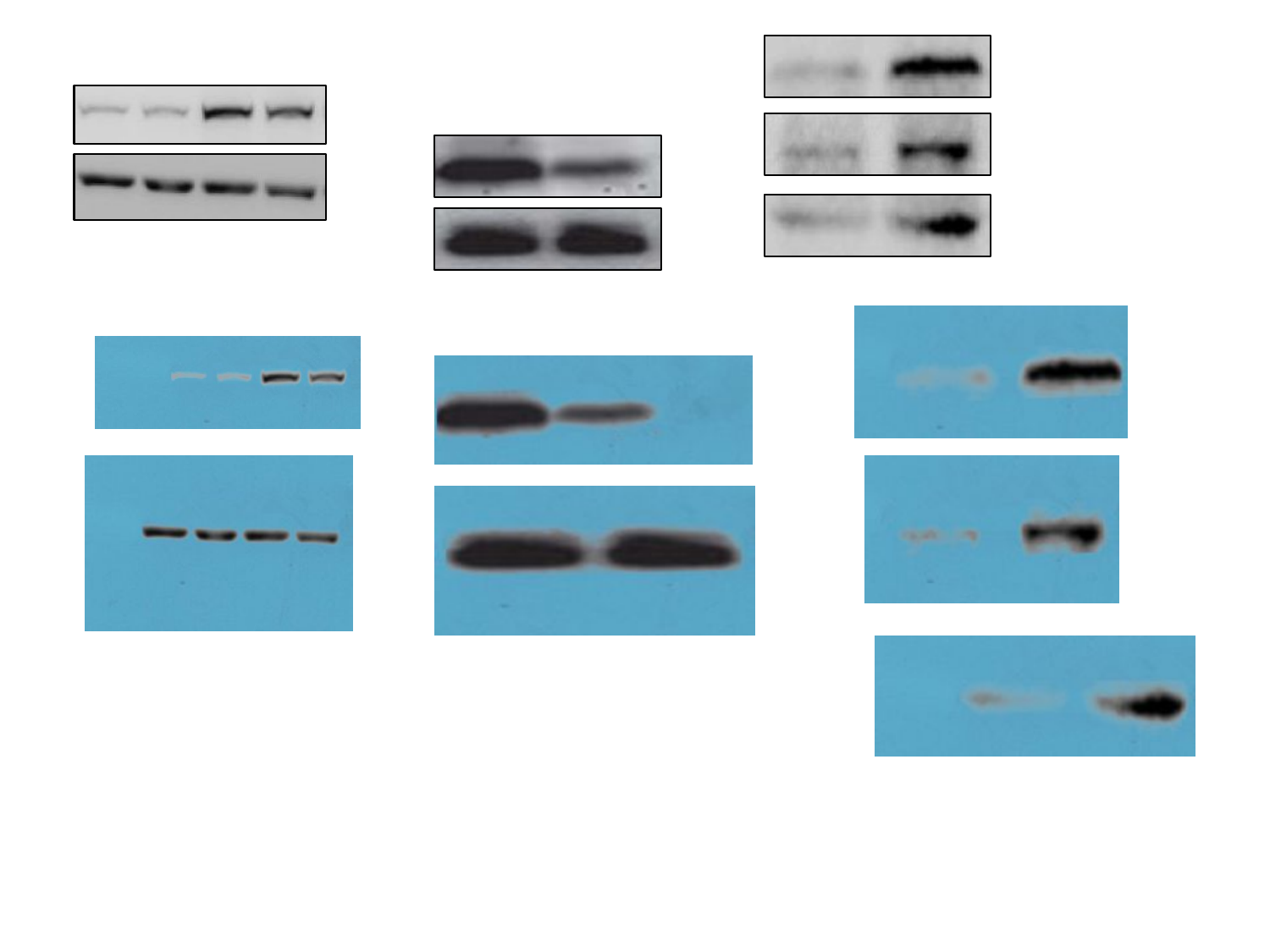

## Slide 4
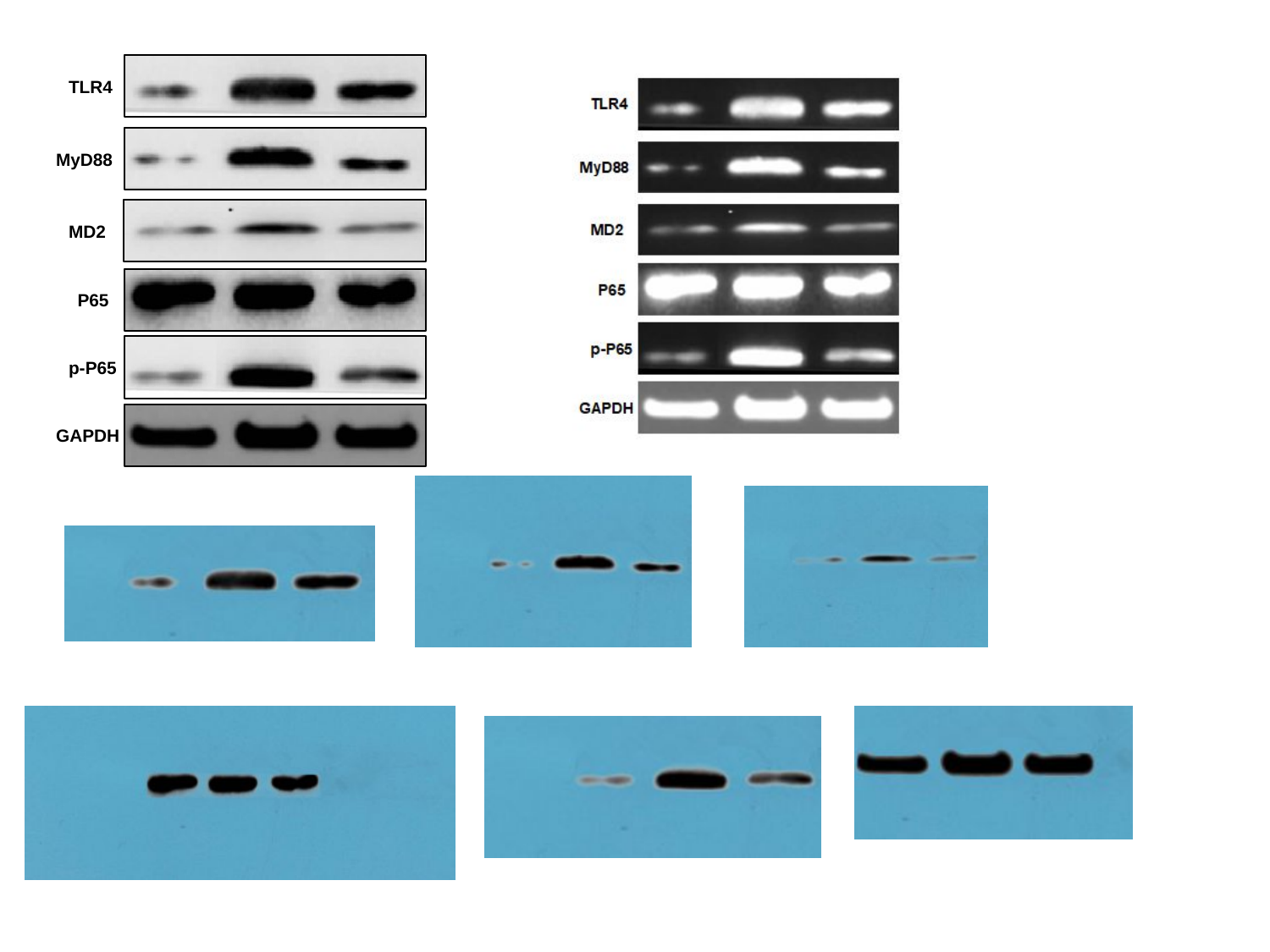

TLR4
MyD88
MD2
P65
p-P65
GAPDH

Supplement: Supplementary file 1 [file Presentation_1.ppt]
